# A microdose cocktail and population pharmacokinetic study suggests potentially reduced CYP3A and P-gp activities in type 2 diabetes

**DOI:** 10.3389/fphar.2026.1924307

**Published:** 2026-08-28

**Authors:** Yafen Li, Jin Yang, Enda Zhou, Kuo Geng, Zihan Lei, Xueting Yao, Feifei Feng, Yuyan Jin, Weijie Kong, Yiyi Hu, Hao Liang, Haiyan Li, Guo-Ping Yang, Tianpei Hong, Dongyang Liu

**Affiliations:** 1 Drug Clinical Trial Center, Peking University Third Hospital, Beijing, China; 2 Department of Endocrinology and Metabolism, Peking University Third Hospital, Beijing, China; 3 Center of Clinical Pharmacology, The Third Xiangya Hospital, Central South University, Changsha, China; 4 Center of Basic Medical Research, Institute of Medical Innovation and Research, Peking University Third Hospital, Beijing, China; 5 Department of Pulmonary and Critical Care Medicine, Peking University Third Hospital, Beijing, China

**Keywords:** CYP3A and transporters, mathematical modeling, microdose cocktail, pharmacokinetics, type 2 diabetes

## Abstract

**Background:**

Diabetes patients often exhibit suboptimal efficacy and adverse reactions, accompanied by pharmacokinetic (PK) changes, indicating drug-metabolizing enzymes and transporters (DMET) activities may change. This study aimed to determine whether type 2 diabetes affects DMET activities and quantify impact magnitude. Furthermore, gut microbiome, pharmacogenetic and demographic characteristics were assessed to elucidate the sources of inter-individual variability (IIV) in DMET activities.

**Methods:**

The activities of CYP3A and transporters P-gp, OATP and BCRP were evaluated in type 2 diabetes patients and healthy volunteers (HVs) following a single oral dose of five probe drugs (midazolam, dabigatran etexilate, pitavastatin, rosuvastatin, and atorvastatin). Population pharmacokinetics (PopPK) and an exploratory machine learning (ML) analysis were employed to quantify the impact of type 2 diabetes on DMET activities.

**Results:**

Based on observed PK parameters, compared to HVs, type 2 diabetes patients exhibited increased exposure to midazolam (1.38-fold), dabigatran (1.40-fold), and atorvastatin (1.93-fold), whereas pitavastatin (0.931-fold) and rosuvastatin (1.25-fold) showed no change. PopPK analysis revealed that the mean activities of CYP3A and P-gp were potentially decreased by 23% and 27%, respectively, in type 2 diabetes patients, while no changes for OATP and BCRP. Notably, unlike the direct patient population covariate for P-gp, reduced CYP3A activity was indirectly estimated from lower *Salmonella* abundance. Additionally, factors such as sex, BCRP genotype, and *Clostridium_XlVb* partly explained the IIV in probe drugs exposure.

**Conclusion:**

The findings regarding altered DMET activities and identified influence factors help to identify specific drug classes that may warrant closer clinical attention in type 2 diabetes patients.

## 1 Introduction

Diabetes affects about 10% of the worldwide population, with more than 90% of cases being type 2 diabetes (Sun et al., 2022). Patients with type 2 diabetes often need multiple agents with different pharmacological actions to treat various complications (Rendell et al., 1983). However, compared to other patients, individuals with type 2 diabetes often show unexpected responses to different drugs in routine clinical practice: easier to experience suboptimal therapeutic outcomes or safety risks (Dostalek et al., 2012; Akhlaghi et al., 2012; Hall et al., 2011). For example, diabetes patients have a significantly higher risk of death, myocardial infarction, and stroke associated with clopidogrel resistance compared to other patients, with the risk increased by 2.92-fold (Steinhubl et al., 2002), and they are more susceptible to atorvastatin (ATV)-induced myotoxicity (Dostalek et al., 2012). In addition, individuals who experienced a major bleeding event when taking dabigatran (DAB) 150 mg twice daily were more likely to have diabetes (89.3% vs. 78.5%) (Aggarwal et al., 2023). This drug response heterogeneity often occurs alongside pharmacokinetic (PK) changes in type 2 diabetes patients (Gravel et al., 2019; Angiolillo et al., 2014). Moreover, some studies suggest that type 2 diabetes impacts the activity of drug-metabolizing enzymes and transporters (DMET) (Li et al., 2024; Gravel et al., 2019; Yao et al., 2020; Liu et al., 2012; Wang et al., 2019).

In DMET, the activities of various cytochrome P450 enzymes (CYP450s) have been demonstrated to change in type 2 diabetes patients, including decreased activities of CYP2C19, CYP3A, and CYP2B6, and increased activity of CYP2C9 (Gravel et al., 2019; Li et al., 2024; Marques et al., 2002). However, these PK studies using CYP450s probe drugs are predominantly limited to Caucasian patients, and the reason for inter-individual variability (IIV) has not been fully elucidated. For transporters, preclinical PK studies in diabetic rat models suggest that diabetes affects the activity of P-glycoprotein (P-gp), organic anion transporting polypeptide (OATP) and breast cancer resistance protein (BCRP) (Zhang et al., 2011; Yao et al., 2020; Wang et al., 2019; Liu et al., 2012), but these findings have not been supported by clinical data, and clinical IIV was unclear. The aforementioned DMET play a crucial role in drug absorption, metabolism, and excretion, and alterations in their activity can significantly impact drug exposure and therapeutic efficacy (Zanger and Schwab, 2013; Lin and Yamazaki, 2003; Niemi et al., 2011; Mao and Unadkat, 2015). Notably, many drugs are disposed by multiple DMET, like clopidogrel, intestinal P-gp limits its absorption, various CYP450s involve the conversion of active metabolite clopidogrel-AM (Clop-AM) (Jiang et al., 2015). The area under the concentration-time curve (AUC) of Clop-AM is reduced by ∼40% in T2DM patients, resulting in unexpected clopidogrel resistance. However, both the activities of CYP450s decrease and the activity of P-gp increase can contribute to this outcome (Angiolillo et al., 2014). Therefore, a comprehensive understanding of the impact of type 2 diabetes on DMET activities is essential for predicting PK changes and minimizing therapeutic variability in type 2 diabetes patients.

Previous studies have demonstrated that gut microbiome composition affects the activity of CYP3A, P-gp and BCRP, as shown by comparisons between germ-free or antibiotic-treated subjects and controls (Jarmusch et al., 2020; Degraeve et al., 2023; González-Sarrías et al., 2013). Moreover, in diabetic rats, the increased expression and activity of P-gp were reversed after antibiotic treatment altered microbiome distribution (Chen et al., 2022). Given the significant changes in gut microbiome composition observed in type 2 diabetes patients compared to healthy individuals (Gurung et al., 2020), it is reasonable to suspect that the gut microbiome contributes to the impact of type 2 diabetes on DMET activities.

The microdose cocktail is a safe and efficient PK study method that enables simultaneous assessment of multiple DMET activities based on the PK of multiple probe drugs. Importantly, the extremely low systemic exposure ensures that no drug-drug interactions (DDI) occur among the co-administered probes (Chavez-Eng et al., 2018; Tatosian et al., 2021). Population pharmacokinetics (PopPK) and machine learning (ML) are powerful tools for identifying and quantifying PK influence factors, while adjusting for clinical confounders. To evaluate the impact of type 2 diabetes on DMET, the microdose cocktail PK study combined with PopPK analysis was performed. Additionally, an ML analysis was conducted as an exploratory approach to supplement the PopPK findings. The cocktail drugs included midazolam (MDZ; CYP3A), dabigatran etexilate (DABE; P-gp), pitavastatin (PTV; OATP), rosuvastatin (RSV; OATP, BCRP), and ATV (CYP3A, OATP, BCRP, and P-gp), which were administered to Chinese type 2 diabetes patients and healthy volunteers (HVs). Additionally, the role of gut microbiome in the impact of type 2 diabetes on DMET was explored through correlation analysis.

## 2 Methods

### 2.1 Participants

Based on the previously reported clinical cocktail studies (Kong et al., 2025; Tatosian et al., 2021), this study planned to enroll 12 type 2 diabetes patients. Type 2 diabetes was diagnosed based on glycated hemoglobin (HbA1c) ≥ 6.5%, fasting blood-glucose ≥7.0 mmol/L, and medical history. To minimize the confounding effects of age, body weight, and hepatic or renal function on DMET activities, strict eligibility criteria were applied to all subjects. Participants were required to be non-smokers, aged 18–65 years, with body mass index (BMI) between 18.5 and 27.9 kg/m^2^, and possessing normal liver and kidney function. Additionally, to prevent food or drug-induced alterations to DMET activities or the gut microbiome, participants were required to abstain from grapefruit juice, antibiotics, probiotics, and CYP450 and transporter inhibitors or inducers for 2 weeks prior to drug administration. This study was conducted in accordance with the Declaration of Helsinki and approved by Peking University Third Hospital medical science research ethics committee. The study protocol was registered at Clinicaltrials.gov (NCT06273215). Data from HVs were obtained from our previously published cocktail study (Kong et al., 2025).

### 2.2 Microdose cocktail procedures

On the evening prior to the study, the mixed probes solution, containing 10 µg MDZ, 375 µg DABE, 10 µg PTV, 50 μg RSV, and 100 μg ATV, was prepared and stored at 4 °C. Detailed preparation method is provided in Supplementary Material. All participants fasted overnight before administration and continued fasting until the 4-h PK time point. Blood samples of HVs were collected before (time 0) and at 0.5, 1, 2, 4, 8, 12, and 24 h after probes administration. For type 2 diabetes patients, to minimize the clinical sampling burden while taking advantage of the PopPK approach for robust PK parameter estimation from sparse data, a time-matched sparse sampling design was implemented for this cohort. Blood samples were specifically collected before (time 0) and at 1, 4, and 24 h after administration. These time points were selected to cover the absorption, distribution, and elimination phases of the five probe drugs to the greatest extent possible. The blood samples were centrifuged to separate the plasma, which was aliquoted into two cryovials and stored at −80 °C until analysis. 1M ammonium acetate (pH 5.0) stabilizer was added to one cryovial to prevent the interconversion between statins and their metabolites (Chavez-Eng et al., 2018).

### 2.3 Bioanalysis

Plasma concentrations of probe drugs were quantified using the validated liquid chromatography-mass spectrometry (LC-MS/MS, Shimadzu) methods. The assay linearity ranges were 1–1,000 pg/mL for statins, 0.5–256 pg/mL for MDZ, and 10–5,120 pg/mL for DAB (the major metabolite of DABE). Concentrations below the lower limit of quantification (LLOQ) (BLQ) were treated as missing values in the data analysis. The precision, accuracy and stability of the assays were within acceptable ranges, could be used to support the PK analysis. As shown in Supplementary Table S1, the intraday and interday precision (CV%) and accuracy (%) for low, medium, and high quality control (QC) were within 15%, except for the LLOQ, which was within ±20%. Under the tested stability conditions, analyte recoveries in QC samples were within 85%–115% of the nominal concentrations (Supplementary Table S2).

### 2.4 PK non-compartmental analysis (NCA)

PK curves for five probe drugs administered to HVs and type 2 diabetes patients were plotted using R (version 4.4.1). PK parameters, including maximum concentration (C_max_) and AUC_0–24 h_, were calculated using the linear-up/log-down method of NCA in Phoenix WinNolin (version 8.3.3).

### 2.5 Gut microbiome

The fecal samples from participants before administration were collected for 16S rRNA gene sequencing of gut microbiome. The V3-V4 hypervariable regions of the bacteria 16S rRNA gene were amplified with primers 338F (5′-ACT CCT ACG GGA GGC AGC AG-3′) and 806R (5′-GGA CTA CHV GGG TWT CTA AT-3′) and sequenced using the MGI-2000 platform (BGI, Shenzhen, China). Bioinformatics analysis was performed to determine the gut microbiome abundance. The correlations between bacterial genus abundance and DMET activities, represented by PopPK-calculated apparent clearance (CL/F), were analyzed using R (version 4.4.1).

### 2.6 Genotyping

Blood samples collected at 0 h were used for DNA extraction. Genotyping was conducted using matrix-assisted laser desorption/ionization time-of flight mass spectrometry (MALDI-TOF MS) provided by MassARRAY from Agena Bioscience. Genotype-predicted phenotypes were determined following the guidance of the Clinical Pharmacogenetics Implementation Consortium (https://www.pharmgkb.org/). The phenotypic analysis of DMET focused on CYP3A5, OATP1B1, and BCRP. CYP3A5 haplotypes were defined based on the rs776746 (219–237 A>G) single nucleotide polymorphisms (SNPs), and the phenotype was categorized as normal (NM), intermediate (IM), or poor metabolizers (PM). OATP1B1 phenotype was classified as normal (NF), decreased (DecF), or poor function (PF) based on the combination of rs2306283 (388G>A) and rs4149056 (521T>C) SNPs, and BCRP phenotype was classified as NF, DecF, or PF based on the rs2231142 (421C>A) SNPs. The impact of phenotypes on DMET activities was analyzed by R (version 4.4.1).

### 2.7 PopPK modeling

Pooling the PK data of HVs and type 2 diabetes patients, and using the first-order conditional estimation with interaction (FOCEI) method of nonlinear mixed-effects modeling (NONMEM) software (version 7.3.0) to develop PopPK model. Different structural models, including various compartment numbers with different absorption (first-order, zero-order, and combined zero- and first-order absorption) (Chatelut et al., 1999) and elimination (linear and nonlinear elimination) (Cai et al., 2020) models, were compared to select the best structural model, based on lowest Bayesian Information Criterion (BIC). The exponential model and proportional model were used to describe the inter-individual variabilities (IIV) and residual variation (RV), respectively.

After identifying structural model for each drug, significant covariates were screened using a stepwise method, with forward inclusion and backward exclusion criteria set at P < 0.05 and P < 0.01, respectively, to assess the impact of type 2 diabetes on the DMET activities. Besides the covariate of disease state (with or without type 2 diabetes) and gut microbiome, the demographic data (e.g., age, body weight) and gene polymorphism were also screened to adjust the potential confounding factors or explore significant influence factors. Goodness of fit (GOF), visual predictive check (VPC), and bootstrap were used to evaluate the final model.

### 2.8 PopPK simulation

The significant covariates related to type 2 diabetes identified by PopPK, such as disease state and differential bacterial genus compared to HVs, were used to generate virtual volunteers for type 2 diabetes and HVs. Meanwhile, demographic and gene polymorphism characteristics were kept same, and PK simulations for 1,000 virtual individuals were performed in each group. The simulated PK parameters for type 2 diabetes patients and HVs were compared to quantify the magnitude of the impact of type 2 diabetes on DMET activity.

### 2.9 ML analysis

The ML modeling workflow was conducted using R (version 4.4.1). Variables with missing values exceeding 50% were excluded and the remaining missing values were handled through multiple imputations. If two continuous variables exhibited significant collinearity (correlation >0.6, and P < 0.05), one of them was excluded the covariate screen. Correlation analysis between individual CL/F and potential confounders was performed to identify covariates with significant correlation (correlation >0.3, and P < 0.05). Important variables were then selected using random forest. 10-fold cross-validation by generalized linear regression was employed to determine the optimal set of variables and establish the quantitative prediction model for individual CL/F. The predictive performance of ML model for CL/F of probe drugs was evaluated by regression fit plot.

### 2.10 Statistical analysis

Pearson correlation coefficients were calculated to evaluate the relationships between continuous covariates, such as CL/F and bacterial genus abundance, with statistical significance assessed using two-tailed tests. Continuous variables were compared between the two study populations or phenotype groups using the Mann-Whitney U test or Student’s t-test, depending on data distribution. Categorical variables were expressed as counts and percentages, and were compared with Fisher’s exact test or Chi-square test.

## 3 Results

### 3.1 Demographic

A total of 13 Chinese type 2 diabetes patients and 14 Chinese HVs successfully completed the study. Table 1 summarizes the demographics and medications of the study participants. Type 2 diabetes patients were slightly older than HVs. Although the type 2 diabetes group had a higher weight and body mass index (BMI) than the HV group, both groups were non-obese according to World Health Organization guidelines. Alanine aminotransferase (ALT) levels were slightly higher in type 2 diabetes patients than in HVs, but all participants had normal liver function. To avoid including participants with impaired liver or renal function, the disease duration of type 2 diabetes patients was relatively short in this study (range: 1.5–8.5 years, mean ± SD: 2.62 ± 1.99 years). Approximately 69% of type 2 diabetes patients were treated with one or more glucose-lowering drugs.

**TABLE 1:** Demographic and clinical characteristics in HV and type 2 diabetes groups.

Parameters (Unit)	HV (N = 14)	Type 2 diabetes (N = 13)	*P* Value
Age (years)	35.64 ± 13.17	49.23 ± 11.58	0.009^a^
Female: Male, no. (%)	10: 4, (71.4%: 28.6%)	8: 5, (61.5%: 38.5%)	0.892
Weight (kg)	58.83 ± 8.54	67.08 ± 9.96	0.037^a^
BMI (kg/m^2^)	22.37 ± 2.59	24.90 ± 2.22	0.015^a^
HbA1c (%)	ND	7.62 ± 0.77	NA
ALT (U/L)	13.07 ± 5.40	25.23 ± 13.92	0.010^a^
AST (U/L)	18.21 ± 5.04	23.77 ± 11.21	0.120
ALB (g/L)	44.91 ± 3.65	45.82 ± 2.61	0.467
Urea (mmol/L)	4.63 ± 0.99	4.72 ± 1.60	0.866
Cr (μmol/L)	70.14 ± 10.41	66.92 ± 11.72	0.457
eGFR (mL/min/1.73 m^2^)	106.64 ± 8.42	103.64 ± 17.86	0.588
LDL (mmol/L)	2.59 ± 0.72	2.95 ± 0.57	0.170
TG (mmol/L)	0.94 ± 0.44	2.27 ± 2.39	0.070
HCT	0.44 ± 0.09	0.44 ± 0.03	0.860
Time since diagnostic (years)	0	2.62 ± 1.99	NA
Medication use, no. (%)	NA	Metformin: 7 (53.8%)	NA
​	​	Acarbose: 2 (15.4%)	​
​	​	Glyburide: 2 (15.4%)	​
​	​	Dapagliflozin: 1 (7.69%)	​

Continuous variables are presented as mean ± SD.

HV, healthy volunteer group; BMI, body mass index; HbA1c, glycated hemoglobin; ALT, alanine aminotransferase; AST, aspartate aminotransferase; ALB, serum albumin; Cr, serum creatinine; eGFR, estimated glomerular filtration rate; LDL, low density lipoprotein; TG, triglyceride; HCT, hematocrit; ND, not detected; NA, not applicable.

^a^ Demographic parameters are significantly different between study groups (*P* < 0.05).

### 3.2 PK comparison

A total of 164 plasma PK samples were collected from all participants, but the quantifications of 4 MDZ samples, 1 DAB sample and four statins samples failed due to inadequate plasma volume. Among the remaining samples, the number of BLQ samples was 20 for MDZ, 3 for DAB, 3 for RSV, 1 for PTV, and 2 for ATV.

**FIGURE 1:**
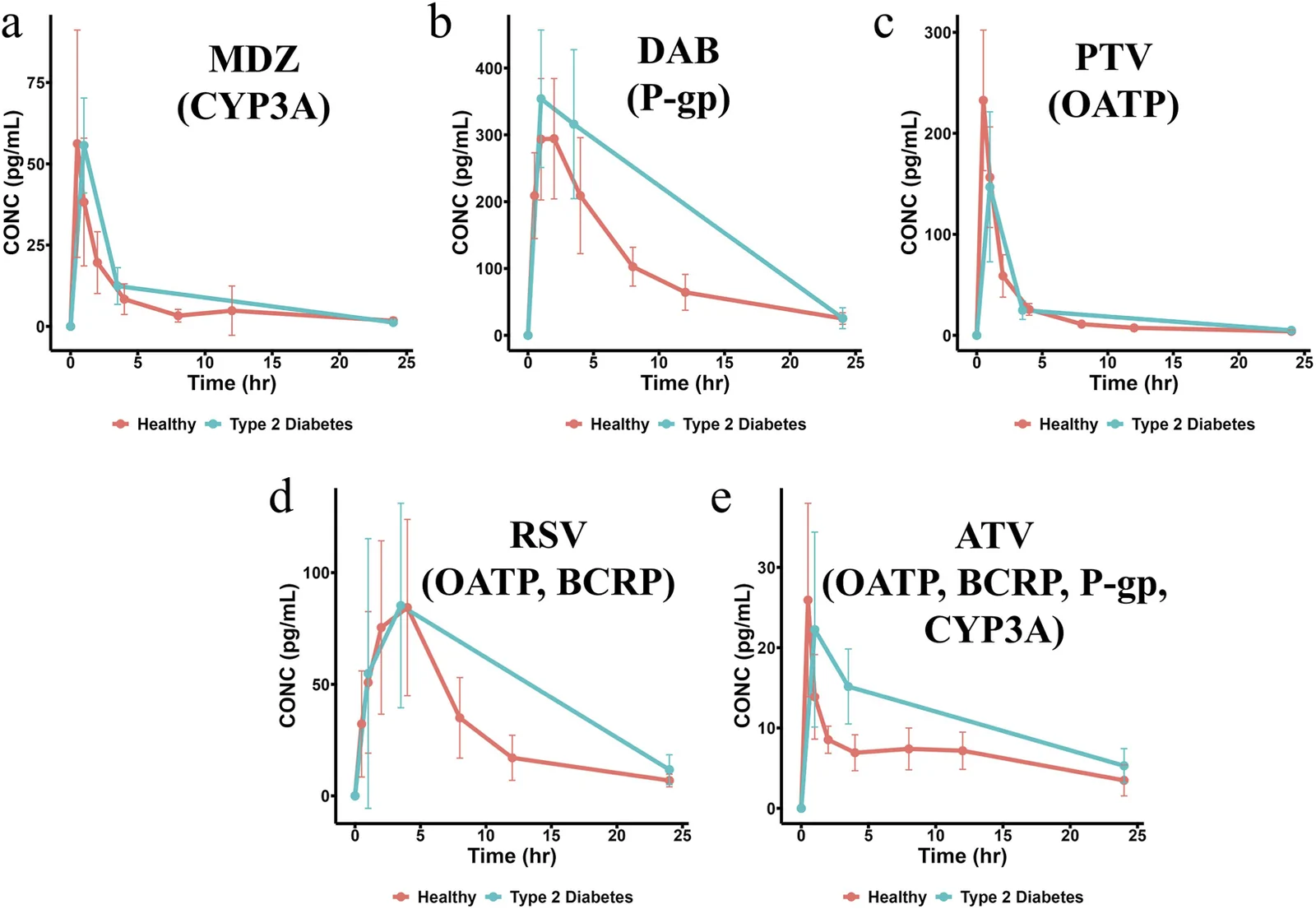
Plasma concentration-time curves of MDZ **(a)**, DAB **(b)**, PTV **(c)**, RSV **(d)**, and ATV **(e)** in healthy volunteers (red) and type 2 diabetes patients (green). The dots represent the mean concentration and the bars are the standard deviation (SD). A sparse sampling design was applied to the type 2 diabetes patients.

The PK curves are shown in Figure 1, which demonstrate that the exposures of DAB and ATV were increased in type 2 diabetes patients compared to HVs. PK parameters were calculated based on the common sampling time points (0, 1, 4, 24 h) for both groups to avoid erroneous PK comparisons due to differing sampling schedules. As shown in Supplementary Table S3, the AUC_0–24h_ of DAB and ATV increased by 40% (*P* = 0.057), and 93% (*P* = 0.004), respectively. For MDZ, the AUC_0–24h_ increased by 38%, but this change did not reach statistical significance (*P* = 0.214). Concurrently, the time-matched C_max_ of MDZ increased significantly by 46% (*P* = 0.016). These results suggest that the activities of CYP3A and P-gp may be reduced in type 2 diabetes patients. However, OATP and BCRP activity were not affected by type 2 diabetes, no change was observed in the AUC_0–24h_ of PTV and RSV (the PK ratios of type 2 diabetes *versus* HV were 0.931 and 1.25, respectively).

### 3.3 PopPK analysis

**FIGURE 2:**
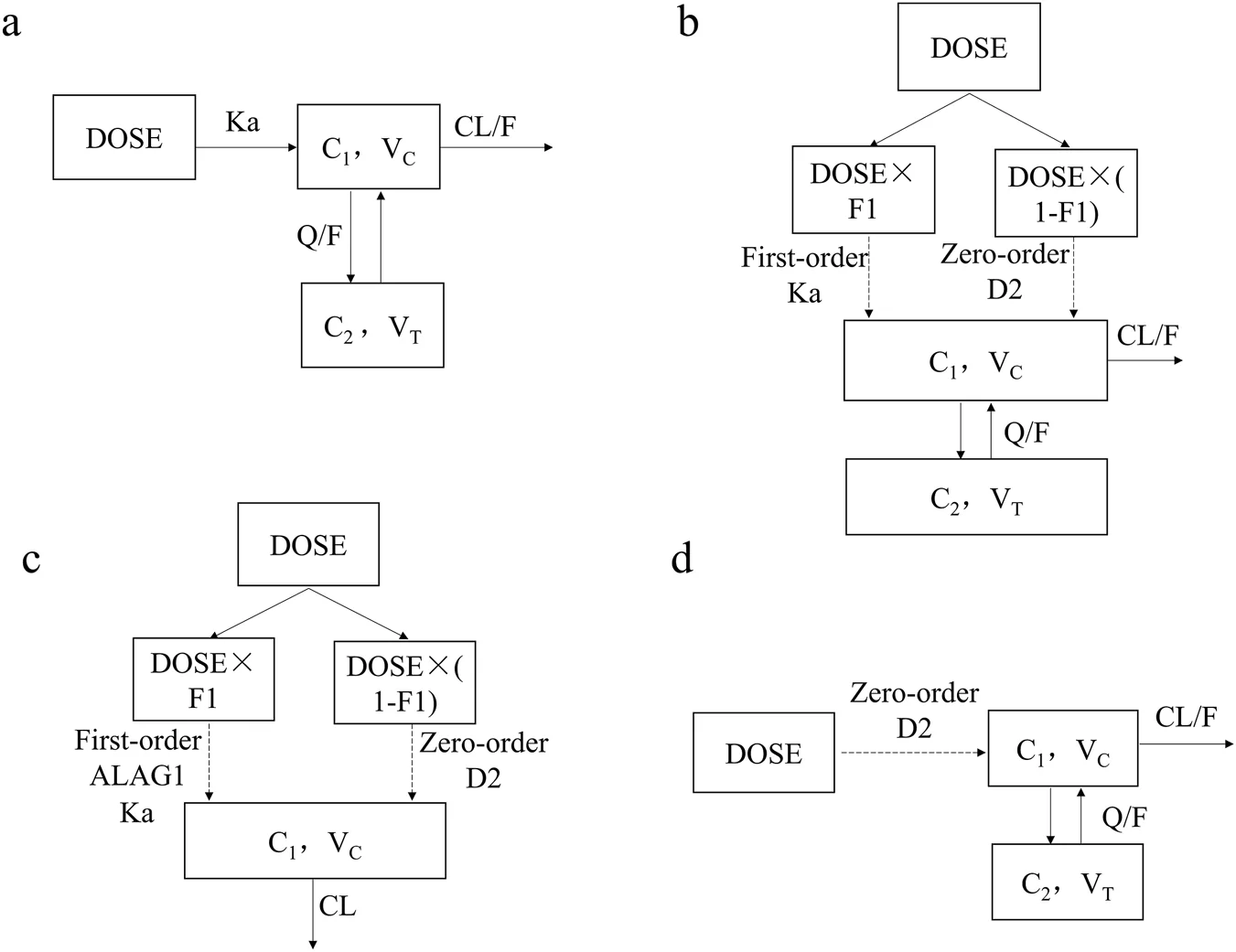
Schematic diagrams of structural models for probe drugs. Among them, the structural model for DAB and MDZ was two-compartment model with first-order absorption **(a)**; for PTV was two-compartment model with combined zero- and first-order absorption **(b)**; for RSV was one-compartment model with combined zero- and first-order absorption **(c)**; for ATV was two-compartment model with zero-order absorption **(d)**. In addition, the elimination models for all drugs were linear **(a–d)**.

All PK concentration data from Type 2 diabetes patients and HVs were included in the PopPK analysis. The most appropriate PopPK structural diagrams for each drug, determined based on the lowest BIC alongside plausible parameter estimates and acceptable condition numbers, are shown in Figure 2. The results of parameters and covariates analysis are presented in Supplementary Table S4 and Equations 1–6. Model performance was effectively validated by GOF, VPC (Supplementary Figures S1 and S2) and bootstrap (Supplementary Table S4). Covariates analysis revealed that type 2 diabetes significantly influenced the PK of DAB and ATV. After accounting for the effect of urea on DAB CL/F (Equation 2), PopPK calculated the relative bioavailability (F) of DAB and ATV in type 2 diabetes patients, compared to HVs was 1.23 (Equation 3) and 1.73 (Equation 6), respectively. The gut microbiome *Salmonella* (Equation 1) and *Clostridium_XlVb* (Equation 5) affected the CL/F of MDZ and RSV, respectively. Moreover, RSV CL/F decreased by 25.9% in participants classified as DecFs of BCRP compared to NFs (Equation 5), the CL/F of PTV in males was 1.42-fold higher than in females (Equation 4).

**FIGURE 3:**
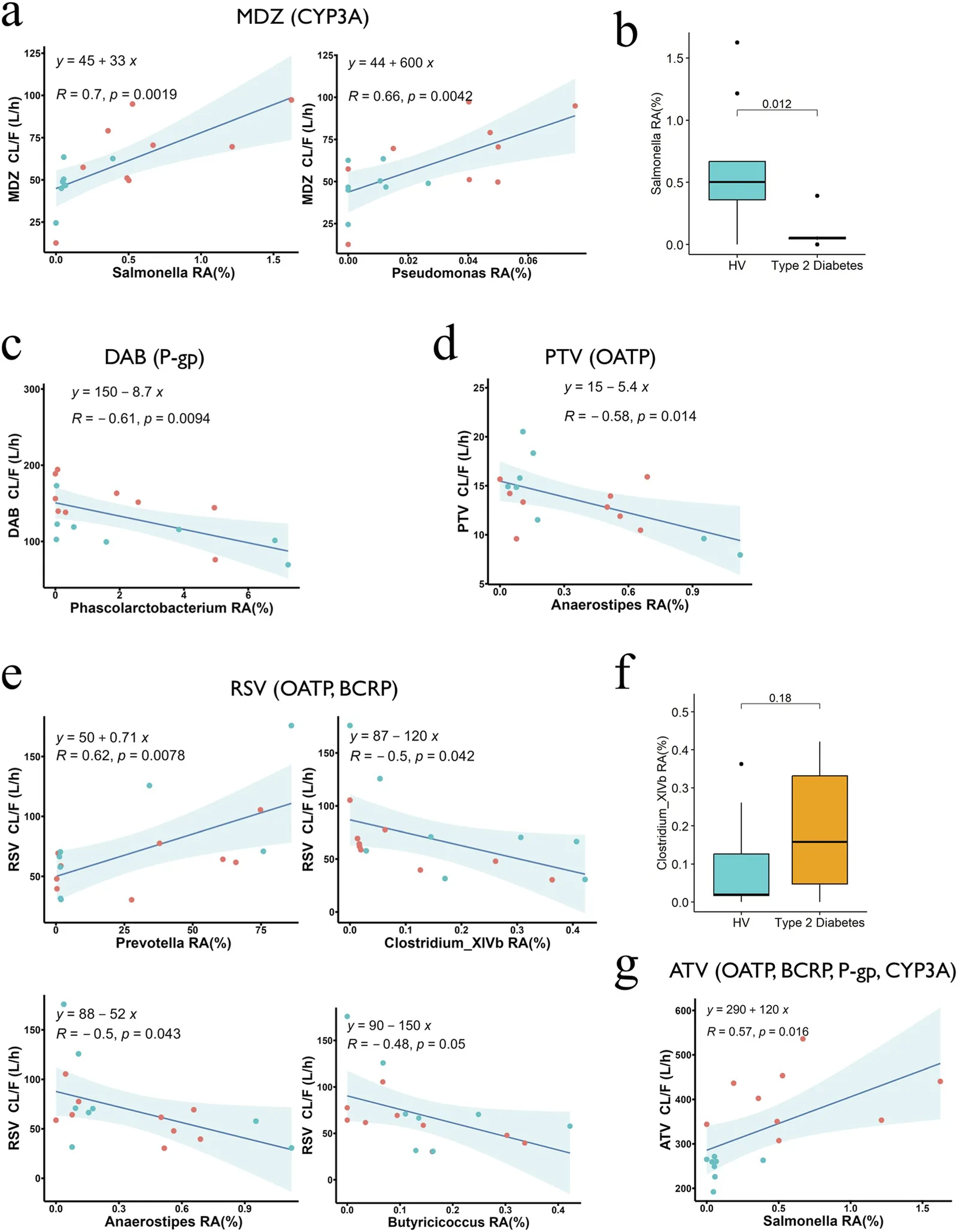
The correlations between specific bacterial genus abundance and CL/F of MDZ **(a)**, DAB **(c)**, PTV **(d)**, RSV **(e)**, and ATV **(g)**, alongside the abundance of *Salmonella* **(b)** and *Clostridium_XlVb* **(f)** in two study populations. The red and green dots in the scatter plots represent the data from healthy volunteers (N = 9) and type 2 diabetes patients (N = 8), respectively. The horizontal lines of box indicate medians and interquartile range (IQR), the error bars of box indicate Q1 minus 1.5 times IQR, and Q3 plus 1.5 times IQR, the black circles indicate outliers. Correlation analyses were performed using Pearson correlation coefficients.

Therefore, P-gp activity might be reduced in type 2 diabetes patients, leading to increased absorption of DAB and ATV. The relative abundance of *Salmonella* was positively correlated with the CYP3A activity, while its abundance was significantly lower in type 2 diabetes patients than in HVs (0.0880 ± 0.124 vs. 0.619 ± 0.505) (Figure 3), indicating CYP3A activity was reduced in type 2 diabetes. Additionally, there was significantly negative relationship between *Clostridium_XlVb* relative abundance and BCRP activity. However, OATP and BCRP activity were not influenced by type 2 diabetes.

1. MDZ (CYP3A):

$$CL/F\left(L/h\right)=57.2\times {\left(\;Salmonella/0.187\right)}^{0.124}$$ (1)

2. DAB (P-gp):

$$CL/F\left(L/h\right)=150\times {\left(\;Urea/4.5\right)}^{-0.749}$$ (2)

$$F=\left\{\begin{array}{c} 1\;\left(if\;HV\right)\\ 1.23\;\left(if\;type\;2\;diabetes\right) \end{array}\right.$$ (3)

3. PTV (OATP):

$$CL/F\left(L/h\right)=\left\{\begin{array}{c} 11.7\;\left(if\;Female\right)\\ 11.7\times 1.42\;\left(if\;Male\right)\; \end{array}\right.$$ (4)

4. RSV (OATP, BCRP):

$$CL/F\left(L/h\right)=\left\{\begin{array}{c} \;66.9\times {\left(Clostridium\_XlVb/0.0628\right)}^{-0.0847}\;\left(if\;BCRP\;NF\right)\\ \;66.9\times {\left(Clostridium\_XlVb/0.0628\right)}^{-0.0847}\times 0.741\;\left(if\;BCRP\;DecF\;\right) \end{array}\right.$$ (5)

5. ATV (OATP, BCRP, P-gp, CYP3A):

$$F=\left\{\begin{array}{c} 1\;\left(if\;HV\right)\\ 1.73\;\left(if\;type\;2\;diabetes\right) \end{array}\right.$$ (6)

### 3.4 Quantifying impact magnitude

The PopPK model showed good predictive performance, with the simulated PK adequately covering the observed data for both populations (Supplementary Figure S3), and the observed PK parameters for HVs closely matched the simulations (Table 2). The simulation results of MDZ CL/F quantitatively revealed that the CYP3A activity was decreased by 23% in type 2 diabetes patients with the low abundance *Salmonella* status. P-gp activity in type 2 diabetes patients was estimated to decrease by 27%, as the simulation value of DAB AUC_0–24h_ increased by 27%. A 42.0% decrease in ATV CL/F in type 2 diabetes patients, which was attributed to the combined reduction in both CYP3A and P-gp activity.

**TABLE 2:** PopPK simulated PK parameters for both groups and the observed PK parameters in HVs.

Probe drugs (DMET)	Parameters (Unit)	HV Obs	HV Sim	Type 2 diabetes Sim	Type 2 diabetes/HV
MDZ (CYP3A)	C_max_ (pg/mL)	56.9 ± 34.4	52.2 ± 10.4	60.3 ± 10.6	1.16
​	AUC_0–24h_ (pg/mL∙h)	135 ± 65.3	136 ± 41.7	170 ± 48.1	1.25
​	CL/F (L/h)	73.6 ± 43.2	75.5 ± 26.0	58.2 ± 20.5	0.771
DAB (P-gp)	C_max_ (pg/mL)	314 ± 88.7	300 ± 55.3	369 ± 68.4	1.23
​	AUC_0–24h_ (pg/mL∙h)	2,290 ± 795	2,198 ± 386	2,782 ± 463	1.27
​	CL/F (L/h)	154 ± 38.5	160 ± 33.8	125 ± 26.0	0.781
PTV (OATP)	C_max_ (pg/mL)	233 ± 68.5	254 ± 88.1	254 ± 88.1	1.00
​	AUC_0–24h_ (pg/mL∙h)	503 ± 107	489 ± 106	489 ± 106	1.00
​	CL/F (L/h)	19.0 ± 5.31	18.9 ± 5.14	18.9 ± 5.14	1.00
RSV (OATP, BCRP)	C_max_ (pg/mL)	83.7 ± 43.0	80.0 ± 35.8	80.0 ± 35.8	1.00
​	AUC_0–24h_ (pg/mL∙h)	661 ± 318	781 ± 326	781 ± 326	1.00
​	CL/F (L/h)	72.7 ± 24.7	63.8 ± 26.1	63.8 ± 26.1	1.00
ATV (OATP, BCRP, P-gp, CYP3A)	C_max_ (pg/mL)	26.4 ± 11.7	21.1 ± 10.9	35.7 ± 19.1	1.69
​	AUC_0–24h_ (pg/mL∙h)	148 ± 47.8	143 ± 29.4	245 ± 52.1	1.71
​	CL/F (L/h)	459 ± 141	426 ± 100	247 ± 56.1	0.580

The observed and simulated PK parameters are presented as mean ± SD.

obs, observed values; Sim, simulated values; type 2 diabetes/HV, ratio of simulated PK parameters for type 2 diabetes patients *versus* HVs.

### 3.5 Impact of gut microbiome

The bacterial genera highly correlated (R > 0.3 and P < 0.05) with the individual CL/F are shown in Figure 3. These genera were considered as potential confounders affecting the activity of DMET and were included in the covariate screening for PopPK. Ultimately, only *Salmonella* and *Clostridium_XlVb* were identified as significant covariates, each influencing the PK of MDZ (CYP3A) and RSV (OATP, BCRP), respectively. Moreover, the relative abundance of *Salmonella* was significantly lower in the type 2 diabetes patients compared to HVs (Figure 3b, *P* = 0.012).

### 3.6 Impact of genotyping

**FIGURE 4:**
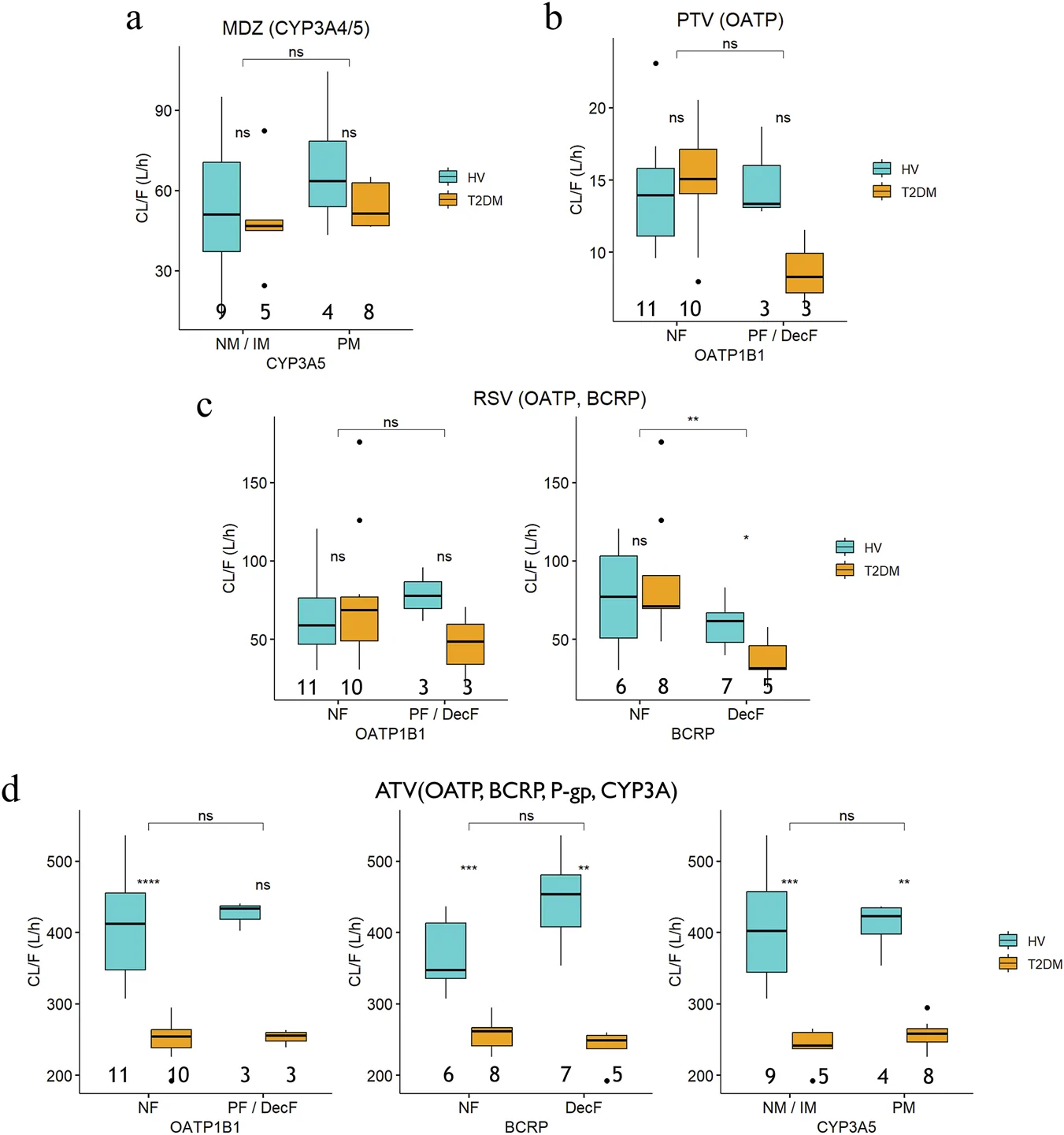
The CL/F of MDZ **(a)**, PTV **(b)**, RSV **(c)** and ATV **(d)** in different phenotype. The horizontal lines of box indicate medians and interquartile range (IQR), the error bars of box indicate Q1 minus 1.5 times IQR, and Q3 plus 1.5 times IQR, the black circles indicate outliers. The numbers of participants in different subgroups were listed at the bottom. The green box and the orange box represent the data in healthy volunteers and type 2 diabetes patients, respectively. (^ns^ no statistical difference; *P < 0.05; **P < 0.01; ***P < 0.001; ****P < 0.0001).

Gene polymorphisms in the two groups are presented in Supplementary Table S5. The NMs and IMs of CYP3A5 were combined, and the PFs and DecFs of OATP were merged into a single phenotype group due to the low incidence. No significant differences were found in the distribution of phenotypes between the two groups (Supplementary Table S6). As shown in Figure 4, the relationships between DMET activity and phenotypes, as well as between DMET activity and type 2 diabetes after adjusting for phenotypes, are depicted. The results suggest that, except for BCRP phenotypes, which significantly affected BCRP activity (with RSV CL/F significantly decreased in BCRP DecF subjects, *P* < 0.01), other genetic phenotypes had no impact on DMET activity. Additionally, across various phenotypes in different genes, ATV CL/F was significantly reduced in type 2 diabetes patients compared to HVs, except for the sample size of OATP1B1 PF/DecF was too small to show statistical differences. An unexpected finding was that type 2 diabetes affected RSV CL/F in BCRP DecFs (*P* < 0.05). Notably, given the small sample sizes within these stratified subgroups, these results remain exploratory.

### 3.7 ML analysis

The important variables affecting the CL/F of probe drugs, as identified by random forest, and the retained variables in final model are shown in Supplementary Figure S4, along with the regression model fit plot in Supplementary Figure S5. The regression equations for each probe drug are provided in Equations 7–11. In addition to the covariates found in the PopPK, ML also accounted for the effects of other bacterial genera, including *Phascolarctobacterium*, *Anaerostipes*, and *Butyricicoccus*, as well as other demographic parameters, such as serum albumin (ALB) and systolic pressure (HBP), on DMET activity.

1. MDZ (CYP3A):

$$CL/F\left(L/h\right)=187-2.99\times ALB+19.0\times Salmonella$$ (7)

2. DAB (P-gp):

$$CL/F\left(L/h\right)=305-22.2\times Urea-8.02\times Phascolarctobacterium-0.712\times HBP+0.932\times Age$$ (8)

3. PTV (OATP):

$$CL/F\left(L/h\right)=14.4-4.73\times Anaerostipes+3.46\times Male$$ (9)

4. RSV (OATP, BCRP):

$$CL/F\left(L/h\;\right)=74.3+9.53\times TG-69.8\times Clostridium\_XlVb-87.5\times Butyricicoccus$$ (10)

5. ATV (OATP, BCRP, P-gp, CYP3A):

$$CL/F\left(L/h\right)=401-152\times type\;2\;diabetes+18.3\times Salmonella$$ (11)

### 3.8 Subgroup analysis of metformin

**FIGURE 5:**
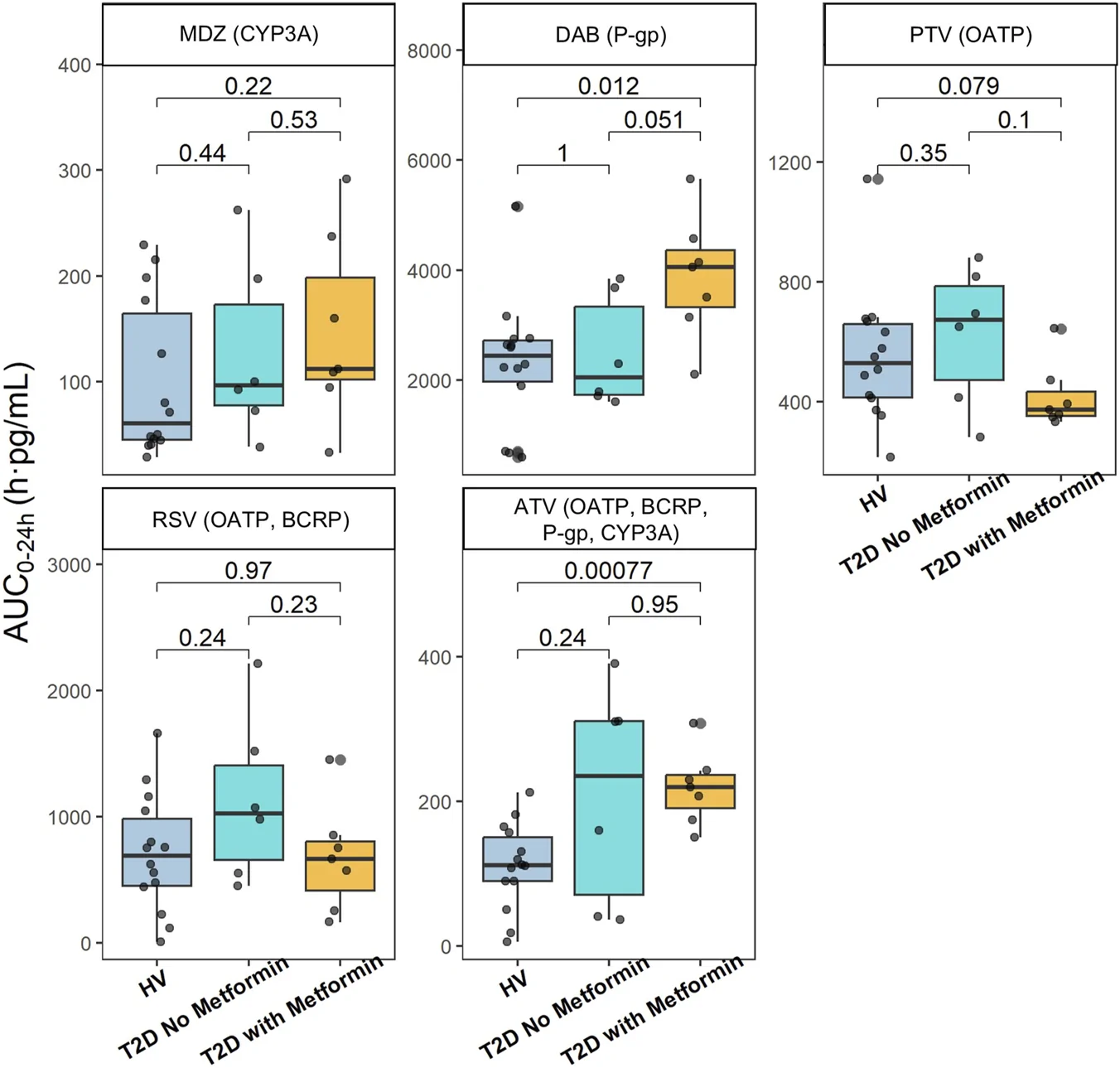
Subgroup analysis of NCA-calculated PK parameters based on whether type 2 diabetes patients were co-administered with metformin. The gray, green, and orange boxes represent healthy volunteers (N = 14), type 2 diabetes patients without metformin (N = 6), and type 2 diabetes patients receiving metformin (N = 7), respectively. The horizontal line within each box indicates the median, the box boundaries represent the interquartile range (IQR), the error bars extend to the lowest and highest values within 1.5 times the IQR.

Given that approximately half of the type 2 diabetes patients were receiving concomitant metformin therapy (Table 1), we conducted a subgroup analysis of the PK parameters for each probe drug based on metformin use. As illustrated in Figure 5, although metformin co-administration did not result in statistically significant changes in AUC_0–24h_ within the type 2 diabetes cohort, trends toward PK alterations were observed. Specifically, compared to those not taking metformin, patients receiving metformin exhibited increased DAB exposure (*P* = 0.051) and decreased PTV exposure (*P* = 0.1). Furthermore, when compared to the HV group, type 2 diabetes patients co-administered with metformin showed statistically significant increases in DAB exposure (*P* = 0.012) and ATV exposure (*P* = 0.00077). The increase in ATV exposure remained significant across the entire type 2 diabetes cohort (Supplementary Table S3, *P* = 0.004), but the increase in DAB exposure did not reach statistical significance (Supplementary Table S3, *P* = 0.057). These results suggest that the elevated DAB exposure observed in type 2 diabetes patients may be partially associated with concomitant metformin use.

## 4 Discussion

By combining the microdose cocktail PK study with PopPK, this study investigated the impact of type 2 diabetes on the activity of four key DMET. Analysis results revealed that gut microbiome status associated with type 2 diabetes decreased CYP3A activity, type 2 diabetes downregulated P-gp activity, but had no impact on the activities of OATP and BCRP. The impact of type 2 diabetes on CYP3A and P-gp transporter resulting in the exposure of MDZ, DAB and ATV increased by 38%, 40%, and 93%, respectively. Additionally, variables such as sex and genotype partly explained the IIV of DMET activity.

Using the probe drug MDZ, PopPK analysis revealed a significant positive correlation between CYP3A activity and *Salmonella* relative abundance (Equation 1). Additionally, *Salmonella* levels were significantly lower in type 2 diabetes patients (Figure 3b), suggesting that CYP3A activity may be reduced in patients. Simulations based on the microbiome characteristics of type 2 diabetes patients calculated a potential 23% decrease in CYP3A activity in Chinese type 2 diabetes patients (Table 2). This indirect estimation exhibits a trend similar to, though milder than, the ∼38% reduction reported in Caucasian type 2 diabetes patients (Gravel et al., 2019; Marques et al., 2002; Dostalek et al., 2011). These findings, alongside previous studies, support the notion that type 2 diabetes reduces CYP3A activity. However, it remains unclear whether this reduction is driven by alterations in the gut microbiome, inflammation, or other factors. Further basic pharmacological studies are needed to explore potential mechanisms.

Contrary to previous findings that OATP and BCRP activity were altered in diabetic rats (Liu et al., 2012; Wang et al., 2019), our clinical study using the probe drugs PTV and RSV found that type 2 diabetes did not affect OATP and BCRP activity. PopPK analysis of PTV revealed that OATP activity was higher in males than in females (Equation 4), which is consistent with a previous study reporting that female subjects had a higher ${\text{AUC}}_{0-\infty }$ of PTV than males (Zhou et al., 2013). Additionally, our exploratory ML analysis found that *Anaerostipes* relative abundance was negatively correlated with OATP activity (Equation 9). PopPK analysis of RSV indicated that *Clostridium_XlVb* and BCRP gene polymorphism impacted BCRP activity (Equation 5). Moreover, *Butyricicoccus* was included in the ML model, explaining partial variability in RSV PK (Equation 10).

P-gp is a critical transporter that limits the oral absorption of drugs such as DABE and ATV(Blech et al., 2008; Dong et al., 2008; Wang et al., 2019; Yoshitomo et al., 2023). Previous findings about how diabetic rats impact P-gp activity were arguable, both the increase and decrease of activity were reported (Yao et al., 2020; Zhang et al., 2011). Using the probe drug DABE, this study suggests that P-gp activity was likely decreased in type 2 diabetes patients (Equation 3), leading to increased absorption of DAB and ATV. However, ATV exposure increased by 71%, much more than DAB (27%) (Table 2), due to type 2 diabetes decreased CYP3A activity, which further reduced CL/F ATV. A negative correlation was observed between urea levels and DAB CL/F (Equation 2), which may be due to elevated urea levels indicating progressive decline in kidney function (Lau and Vaziri, 2017), leading to DAB renal excretion rate reduction. Furthermore, the exploratory ML analysis identified that *Phascolarctobacterium* relative abundance was negatively correlated with DAB CL/F (Equation 8), and *Salmonella* was positively correlated with ATV CL/F (Equation 11).

PopPK successfully estimated individual PK parameters for each participant under a sparse sampling strategy in type 2 diabetes patients, thereby enhancing both safety and efficiency of cocktail study (Destere et al., 2023). The significant covariates identified by PopPK were included in the ML model, except for the impact of BCRP phenotype on RSV PK and type 2 diabetes on DAB PK. However, some covariates identified by ML analysis were backward excluded by PopPK, indicating that PopPK performed more stringent criteria for determining factors influencing DMET activity. The advantage of PopPK lies in its ability to prevent multicollinearity between variables and accurately identify clinically significant covariate, but the process is cumbersome (Huang et al., 2025). In contrast, ML offers a faster method for identifying influential factors from complex data, but its interpretability is limited, as it does not incorporate PK disposition information like PopPK does (Valderrama et al., 2025).

Type 2 diabetes patients enrolled in this study were older and heavier than HVs (Table 1). Although PopPK analysis effectively avoided for the impact of confounders on DMET activity, the sensitivity analysis was conducted to further validate the reliability of our findings. After balancing the age and body weight between the two groups, the significant differences were no longer observed (Supplementary Table S7). Sensitivity analysis results were consistent with primary analysis, with exposure of DAB and ATV increased in type 2 diabetes patients (Supplementary Figure S6). For PK parameters, the C_max_ of MDZ, the AUC_0–24h_ of DAB and ATV were increased by 38%, 45% and 65%, respectively, which was similar to the 46%, 40%, and 93% values observed in primary analysis (Supplementary Table S8). The PopPK sensitivity analysis estimated relative F of DAB and ATV in type 2 diabetes patients to be 1.26 and 1.72 times that of HVs, respectively, which closely matched the primary analysis estimates of 1.23 and 1.73. The relative abundance of *Salmonella* was significantly positively correlated with MDZ CL/F (Supplementary Figure S7, R = 0.67), though it did not meet the inclusion criteria for PopPK covariates. Similarly to primary analysis, PopPK sensitivity analysis for RSV included only BCRP phenotype and *Clostridium_XlVb* as significant covariates, with a 38.8% decrease in CL/F in DecFs of BCRP compared to NFs. The relative abundance of *Clostridium_XlVb* was negatively correlated with RSV CL/F (Supplementary Figure S7, R = −0.38).

Because half of the enrolled type 2 diabetes patients were concurrently treated with metformin (Table 1), we performed an exploratory subgroup analysis. Our results (Figure 5) indicate that the increased DAB exposure in type 2 diabetes patients predominantly occurred in the subgroup receiving metformin. This suggests that the elevated DAB exposure might be linked to a DDI with metformin, although the underlying mechanism remains unclear. Furthermore, due to the limited sample size of the current study, it is difficult to definitively distinguish whether the increased DAB exposure is primarily driven by the pathological factors of diabetes itself or by metformin-mediated interactions. Future investigations involving larger type 2 diabetes cohorts are necessary to validate these findings.

Efforts have recently been made to investigate the underlying reasons for ∼40% reduction in Clop-AM AUC in type 2 diabetes patients (Angiolillo et al., 2014). A study in diabetic rats suggested that increased P-gp expression and activity in diabetes reduced clopidogrel absorption, which could be a key factor (Chen et al., 2022). However, this current study found that P-gp activity is actually decreased in type 2 diabetes patients. Therefore, the reduced activity of CYP3A and CYP2C19 in type 2 diabetes patients (Gravel et al., 2019; Li et al., 2024), leading to decreased formation of Clop-AM, more likely to be the primary cause.

This study has some limitations. The composition of the gut microbiome is highly complex, with over 100 bacterial genera detected, and significant correlations were observed between different bacterial genera (results not shown). These interaction between different bacterial genera introduce the possibility of false positive or false negative in the screening of bacterial genus. Therefore, the associations between the genera *Salmonella* and *Clostridium_XlVb* and DMET activity identified in the PopPK analysis are based on data-driven selection. The underlying mechanistic and causal relationships between the pathophysiological states of the human body (such as chronic inflammatory or hyperglycemic conditions), the gut microbiota, and DMET activity remain unclear. Consequently, these findings require further validation through both preclinical animal models and larger-scale clinical studies that incorporate more comprehensive pathophysiological indicators. Another limitation is that the pathological status of type 2 diabetes is complex, whereas the sample size of current study is relatively small. Consequently, our findings regarding the impact of type 2 diabetes on DMET activities may be susceptible to unmeasured confounding factors, highlighting the need to confirm these results in future studies with larger patient populations.

## 5 Conclusion

Based on the primary PopPK analysis, our results indicate that type 2 diabetes may be associated with a 27% reduction in P-gp activity, whereas type 2 diabetes-associated microbiome features are linked to an estimated 23% decrease in CYP3A activity. This study provides additional Chinese data on the impact of type 2 diabetes on CYP3A enzyme activity, and it is the first human study to report the impact of type 2 diabetes on transporter activity, to the best of our knowledge. As more high-quality research on the effects of type 2 diabetes on DMET activity emerges, these findings will help better explain or predict the therapeutic heterogeneity observed in type 2 diabetes patients, mitigate safety risks, and improve therapeutic efficacy.

## Data Availability

The datasets presented in this study can be found in online repositories. The names of the repository/repositories and accession number(s) can be found in the article/Supplementary Material.
