# Supplementary material for: A microdose cocktail and population pharmacokinetic study suggests potentially reduced CYP3A and P-gp activities in type 2 diabetes
Source: Front Pharmacol. 2026 Aug 28;17:1924307. doi: 10.3389/fphar.2026.1924307 (PMC13561922; doi:10.3389/fphar.2026.1924307)
Supplement: Supplementary file 1 [file Supplementaryfile1.docx]

***Supplementary Material***

**Supplementary Methods**

**Microdose cocktail regimen preparation**

MDZ solution (Jiangsu Nhwa Pharmaceutical Co., LTD, 5 mg/mL), DABE capsule (Boehringer Ingelheim International GmbH, 110 mg), PTV tablet (China Resources Double-Crane Pharmaceutical Co., Ltd, 2 mg), RSV tablet (Astra Zeneca Pharmaceutical Co., Ltd, 10 mg) and ATV tablet (Pfizer Inc. 20 mg) were provided by Peking University Third Hospital. Each tablet or capsule content was ground into powder separately. The powders of DABE, PTV, RSV and ATV were dissolved into sterile saline solution in volumes of 110, 100, 100 and 100 mL, respectively. For MDZ, 0.5 mL of the solution was extracted and then diluted with saline to concentration of 0.05 mg/mL. Finally, the parent solutions of MDZ, DABE, PTV, RSV and ATV (0.2, 0.375, 0.5, 0.5 and 0.5 mL, respectively) were taken and diluted with 50 mL of saline.

**Figure S1.** Goodness-of-fit (GOF) plots for the PopPK model of MDZ (a), DAB (b), PTV (c), RSV (d) and ATV (e). The red dotted line represents the fit of a linear regression through the data, with the blue circles representing the observed data.

**Figure S2.** Visual predictive checks (VPC) for the PopPK model of MDZ (a), DAB (b), PTV (c), RSV (d) and ATV (e). The black circle represents observed PK data. The black and red solid line represents the 50th percentile for the observed and simulated data, respectively. The black dotted line lines represent the 5th and 95th percentiles for the observed data. The green dotted lines represent the 5th and 95th percentiles for the simulated data. The shaded areas represent the 90% confidence intervals for the 5th, 50th, and 95th percentiles of the simulated data.

**Figure S3.** The simulated and observed PK curves for MDZ (a), DAB (b), PTV (c), RSV (d) and ATV (e) in HVs and type 2 diabetes patients. The dots and the bars represent the mean and standard deviation of observed PK data. The red and green lines indicate the predicted mean PK data of HVs and type 2 diabetes patients. The red-shaded and green-shaded regions are the 90% prediction intervals of PK data for HVs and type 2 diabetes patients, respectively.

**Figure S4.** The important variables selected by random forest (left) and the retained variables in final ML model (right) for each probe drug.

**Figure S5.** Regression fit plot for ML model of MDZ (a), DAB (b), PTV (c), RSV (d) and ATV (e). The Obs CL/F represents calculated CL/F by PopPK model and the Pred CL/F represents predicted CL/F by ML model.

**Figure S6.** After balancing the age and body weight between the two groups, the plasma concentration-time curves of MDZ (a), DAB (b), PTV (c), RSV (d), and ATV (e) in healthy volunteers (red) and type 2 diabetes patients (green). The dots represent the mean concentration and the bars are the standard deviation (SD).

**Figure S7.** After balancing the age and body weight between the two groups, the correlations between PopPK model calculated CL/F of MDZ and *Salmonella* relative abundance (left), and the correlations between PopPK model calculated CL/F of RSV and *Clostridium_XlVb* relative abundance (right). The red dots and the green dots represent the data in healthy volunteers and type 2 diabetes patients, respectively.

**Table S1.** Precision and accuracy test of all five drugs.

| **MDZ** | | | | | |
| --- | --- | --- | --- | --- | --- |
| Variable | | LLOQ | Low QC | Medium QC | High QC |
| Nominal Concentration (pg/mL) | | 0.5 | 1.5 | 20.0 | 200.0 |
| Intra-day test batch 1  (n = 6) | Mean | 0.5 | 1.5 | 19.6 | 186.4 |
|  | Precision (CV) (%) | 11.7 | 13.4 | 5.6 | 2.7 |
|  | Accuracy (%) | 4.2 | -0.6 | -2.2 | -6.8 |
| Intra-day test batch 2  (n = 6) | Mean | 0.6 | 1.4 | 20.3 | 212.5 |
|  | Precision (CV) (%) | 11.5 | 8.0 | 10.4 | 11.0 |
|  | Accuracy (%) | 16.9 | -9.1 | 1.5 | 6.2 |
| Intra-day test batch 3  (n = 6) | Mean | 0.5 | 1.5 | 21.1 | 188.5 |
|  | Precision (CV) (%) | 8.5 | 7.1 | 9.4 | 4.2 |
|  | Accuracy (%) | 0.3 | -1.9 | 5.6 | -5.7 |
| Inter-day test  (n = 18) | Mean | 0.5 | 1.4 | 20.3 | 195.8 |
|  | Precision (CV) (%) | 12.3 | 9.9 | 8.9 | 9.3 |
|  | Accuracy (%) | 7.5 | -4.1 | 1.6 | -2.1 |
| **DAB** | | | | | |
| Variable | | LLOQ | Low QC | Medium QC | High QC |
| Nominal Concentration (pg/mL) | | 10.0 | 30.0 | 400.0 | 4000.0 |
| Intra-day test batch 1  (n = 6) | Mean | 10.8 | 27.8 | 398.0 | 4262.0 |
|  | Precision (CV) (%) | 15.0 | 5.3 | 3.0 | 2.7 |
|  | Accuracy (%) | 8.0 | -7.3 | -0.5 | 6.6 |
| Intra-day test batch 2  (n = 6) | Mean | 9.9 | 30.2 | 390.6 | 4021.9 |
|  | Precision (CV) (%) | 14.0 | 9.1 | 4.5 | 3.0 |
|  | Accuracy (%) | -0.7 | 0.7 | -2.3 | 0.5 |
| Intra-day test batch 3  (n = 6) | Mean | 10.3 | 31.5 | 394.9 | 4019.3 |
|  | Precision (CV) (%) | 7.9 | 5.1 | 3.8 | 2.9 |
|  | Accuracy (%) | 3.1 | 5.0 | -1.3 | 0.5 |
| Inter-day test  (n = 18) | Mean | 10.3 | 29.8 | 394.5 | 4101.1 |
|  | Precision (CV) (%) | 12.5 | 8.3 | 3.6 | 3.9 |
|  | Accuracy (%) | 3.5 | -0.5 | -1.4 | 2.5 |
| **PTV** | | | | | |
| Variable | | LLOQ | Low QC | Medium QC | High QC |
| Nominal Concentration (pg/mL) | | 1.0 | 3.0 | 48.0 | 800.0 |
| Intra-day test batch 1  (n = 6) | Mean | 1.1 | 2.7 | 44.0 | 702.2 |
|  | Precision (CV) (%) | 17.6 | 9.5 | 4.1 | 8.7 |
|  | Accuracy (%) | 6.7 | -10.1 | -8.3 | -12.2 |
| Intra-day test batch 2  (n = 6) | Mean | 1.1 | 3.0 | 47.5 | 821.4 |
|  | Precision (CV) (%) | 9.7 | 9.8 | 2.7 | 2.0 |
|  | Accuracy (%) | 11.3 | -0.7 | -1.1 | 2.7 |
| Intra-day test batch 3  (n = 6) | Mean | 0.9 | 2.9 | 52.0 | 864.3 |
|  | Precision (CV) (%) | 11.3 | 5.7 | 0.9 | 1.8 |
|  | Accuracy (%) | -7.7 | -2.7 | 8.4 | 8.0 |
| Inter-day test  (n = 18) | Mean | 1.0 | 2.9 | 47.9 | 795.9 |
|  | Precision (CV) (%) | 15.0 | 9.1 | 7.7 | 9.9 |
|  | Accuracy (%) | 3.4 | -4.5 | -0.3 | -0.5 |
| **RSV** | | | | | |
| Variable | | LLOQ | Low QC | Medium QC | High QC |
| Nominal Concentration (pg/mL) | | 1.0 | 3.0 | 48.0 | 800.0 |
| Intra-day test batch 1  (n = 6) | Mean | 1.1 | 2.9 | 46.7 | 772.4 |
|  | Precision (CV) (%) | 19.4 | 8.2 | 7.4 | 2.3 |
|  | Accuracy (%) | 9.3 | -2.7 | -2.7 | -3.4 |
| Intra-day test batch 2  (n = 6) | Mean | 0.9 | 2.8 | 48.5 | 865.3 |
|  | Precision (CV) (%) | 11.7 | 8.9 | 1.6 | 1.4 |
|  | Accuracy (%) | -9.0 | -6.1 | 1.1 | 8.2 |
| Intra-day test batch 3  (n = 6) | Mean | 1.1 | 3.1 | 50.0 | 867.8 |
|  | Precision (CV) (%) | 16.4 | 11.4 | 4.5 | 2.1 |
|  | Accuracy (%) | 14.8 | 3.7 | 4.2 | 8.5 |
| Inter-day test  (n = 18) | Mean | 1.1 | 2.9 | 48.4 | 835.2 |
|  | Precision (CV) (%) | 18.6 | 10.1 | 5.7 | 5.8 |
|  | Accuracy (%) | 5.0 | -1.7 | 0.9 | 4.4 |
| **ATV** | | | | | |
| Variable | | LLOQ | Low QC | Medium QC | High QC |
| Nominal Concentration (pg/mL) | | 1.0 | 3.0 | 48.0 | 800.0 |
| Intra-day test batch 1  (n = 6) | Mean | 1.1 | 3.1 | 44.6 | 685.2 |
|  | Precision (CV) (%) | 13.0 | 11.3 | 8.2 | 7.2 |
|  | Accuracy (%) | 10.8 | 4.1 | -7.1 | -14.4 |
| Intra-day test batch 2  (n = 6) | Mean | 1.1 | 3.2 | 50.6 | 850.5 |
|  | Precision (CV) (%) | 17.2 | 11.2 | 3.0 | 4.8 |
|  | Accuracy (%) | 9.2 | 8.1 | 5.4 | 6.3 |
| Intra-day test batch 3  (n = 6) | Mean | 1.1 | 3.2 | 49.7 | 855.7 |
|  | Precision (CV) (%) | 17.8 | 8.5 | 1.8 | 3.2 |
|  | Accuracy (%) | 6.8 | 5.3 | 3.6 | 7.0 |
| Inter-day test  (n = 18) | Mean | 1.1 | 3.2 | 48.2 | 797.1 |
|  | Precision (CV) (%) | 15.2 | 9.9 | 7.4 | 11.3 |
|  | Accuracy (%) | 8.9 | 5.8 | 0.3 | -0.4 |

**Table S2.** Stability tests of all five drugs.

| **MDZ** | | | | |
| --- | --- | --- | --- | --- |
| Variable | | Low QC | Medium QC | High QC |
| Nominal Concentration (pg/mL) | | 1.5 | 20.0 | 200.0 |
| On-bench stability  (RT for 4 h)  (n = 6) | Mean | 1.5 | 18.5 | 183.5 |
|  | Recovery (%) | 100.3 | 92.4 | 91.7 |
|  | Precision (CV) (%) | 4.7 | 2.8 | 0.6 |
| On-machine stability  (2℃ - 8℃ for 24 h)  (n = 6) | Mean | 1.5 | 18.5 | 181.0 |
|  | Recovery (%) | 100.8 | 92.7 | 90.5 |
|  | Precision (CV) (%) | 7.6 | 5.6 | 2.7 |
| Freeze-thaw stability  (3 cycles)  (n = 6) | Mean | 1.4 | 19.3 | 190.8 |
|  | Recovery (%) | 94.7 | 96.7 | 95.4 |
|  | Precision (CV) (%) | 4.2 | 7.3 | 9.1 |
| **DAB** | | | | |
| Variable | | Low QC | Medium QC | High QC |
| Nominal Concentration (pg/mL) | | 30.0 | 400.0 | 4000.0 |
| On-bench stability  (RT for 4 h)  (n = 6) | Mean | 27.7 | 396.4 | 4134.7 |
|  | Recovery (%) | 92.2 | 99.1 | 103.4 |
|  | Precision (CV) (%) | 3.6 | 4.6 | 3.2 |
| On-machine stability  (2℃ - 8℃ for 24 h)  (n = 6) | Mean | 29.4 | 413.9 | 4349.7 |
|  | Recovery (%) | 98.1 | 103.5 | 108.7 |
|  | Precision (CV) (%) | 3.5 | 2.7 | 2.4 |
| Freeze-thaw stability  (3 cycles)  (n = 6) | Mean | 31.9 | 398.1 | 4223.1 |
|  | Recovery (%) | 106.3 | 99.5 | 105.6 |
|  | Precision (CV) (%) | 7.3 | 6.7 | 4.7 |
| **PTV** | | | | |
| Variable | | Low QC | Medium QC | High QC |
| Nominal Concentration (pg/mL) | | 3.0 | 48.0 | 800.0 |
| On-bench stability  (RT for 4 h)  (n = 6) | Mean | 2.9 | 52.8 | 824.7 |
|  | Recovery (%) | 97.7 | 110.0 | 103.1 |
|  | Precision (CV) (%) | 9.4 | 3.9 | 3.1 |
| On-machine stability  (2℃ - 8℃ for 24 h)  (n = 6) | Mean | 3.2 | 48.9 | 840.8 |
|  | Recovery (%) | 105.2 | 101.8 | 105.1 |
|  | Precision (CV) (%) | 3.1 | 3.0 | 1.3 |
| Freeze-thaw stability  (3 cycles)  (n = 6) | Mean | 3.3 | 50.0 | 882.5 |
|  | Recovery (%) | 110.3 | 104.1 | 110.3 |
|  | Precision (CV) (%) | 3.4 | 1.6 | 1.7 |
| **RSV** | | | | |
| Variable | | Low QC | Medium QC | High QC |
| Nominal Concentration (pg/mL) | | 3.0 | 48.0 | 800.0 |
| On-bench stability  (RT for 4 h)  (n = 6) | Mean | 2.8 | 51.8 | 840.1 |
|  | Recovery (%) | 93.5 | 108.0 | 105.0 |
|  | Precision (CV) (%) | 10.4 | 4.5 | 2.5 |
| On-machine stability  (2℃ - 8℃ for 24 h)  (n = 6) | Mean | 3.1 | 47.4 | 769.0 |
|  | Recovery (%) | 103.8 | 98.7 | 96.1 |
|  | Precision (CV) (%) | 8.5 | 4.7 | 5.8 |
| Freeze-thaw stability  (3 cycles)  (n = 6) | Mean | 2.9 | 50.1 | 874.6 |
|  | Recovery (%) | 97.0 | 104.4 | 109.3 |
|  | Precision (CV) (%) | 7.8 | 4.9 | 2.2 |
| **ATV** | | | | |
| Variable | | Low QC | Medium QC | High QC |
| Nominal Concentration (pg/mL) | | 3.0 | 48.0 | 800.0 |
| On-bench stability  (RT for 4 h)  (n = 6) | Mean | 3.0 | 54.2 | 887.7 |
|  | Recovery (%) | 99.7 | 112.9 | 111.0 |
|  | Precision (CV) (%) | 11.2 | 5.0 | 7.7 |
| On-machine stability  (2℃ - 8℃ for 24 h)  (n = 6) | Mean | 2.8 | 54.4 | 852.4 |
|  | Recovery (%) | 94.3 | 113.3 | 106.5 |
|  | Precision (CV) (%) | 6.3 | 1.1 | 6.4 |
| Freeze-thaw stability  (3 cycles)  (n = 6) | Mean | 3.1 | 52.3 | 839.4 |
|  | Recovery (%) | 103.7 | 109.0 | 104.9 |
|  | Precision (CV) (%) | 7.5 | 6.8 | 5.9 |

RT: room temperature

**Table S3.** PK parameters were calculated at the intersecting time points between the HV and type 2 diabetes groups.

| **Probe drugs (DMET)** | **Parameters (Unit)** | **HV (N=14)** | **Type 2 diabetes (N=13)** | **Type 2 diabetes/HV** | ***P* value** |
| --- | --- | --- | --- | --- | --- |
| MDZ (CYP3A) | C_max_ (pg/mL) | 38.2 (51.4%) | 55.7 (26.2%) | 1.46 | 0.016* |
|  | AUC_0-24 h_ (h·pg/mL) | 99.7 (74.1%) | 138 (60.7%) | 1.38 | 0.214 |
| DAB (P-gp) | C_max_ (pg/mL) | 297 (29.1%) | 361 (29.5%) | 1.22 | 0.102 |
|  | AUC_0-24 h_ (h·pg/mL) | 2309 (50.8%) | 3242 (38.8%) | 1.40 | 0.057^#^ |
| PTV (OATP) | C_max_ (pg/mL) | 157 (31.8%) | 147 (50.5%) | 0.936 | 0.694 |
|  | AUC_0-24 h_ (h·pg/mL) | 551 (39.9%) | 513 (38.9%) | 0.931 | 0.647 |
| RSV (OATP, BCRP) | C_max_ (pg/mL) | 79.5 (60.0%) | 81.2 (75.1%) | 1.02 | 0.932 |
|  | AUC_0-24 h_ (h·pg/mL) | 709 (65.5%) | 886 (64.4%) | 1.25 | 0.386 |
| ATV (OATP, BCRP, P-gp, CYP3A) | C_max_ (pg/mL) | 13.9 (37.9%) | 22.7 (51.4%) | 1.63 | 0.023* |
|  | AUC_0-24 h_ (h·pg/mL) | 111 (53.1%) | 214 (48.6%) | 1.93 | 0.004* |

PK parameters are presented as mean (CV%). *PK parameters are significantly different between study groups (*P* < 0.05). ^#^*P* < 0.1. DMET, drug-metabolic enzymes and transporters; Type 2 diabetes/HV: ratio of PK parameters for type 2 diabetes patients versus HVs.

**Table S4.** PopPK model parameter estimates and bootstraps results.

| **Parameters (Unit)** | | **Estimate (RSE%)** | **Shrinkage** | **Bootstrap** | |
| --- | --- | --- | --- | --- | --- |
|  |  |  |  | **Median** | **90% CI** |
| **1) MDZ (CYP3A)** | | | | Minimization successful: 89% | |
| **structural model** | CL/F (L/h) | 57.2 (9.40%) | - | 56.5 | (39.5, 65.0) |
|  | V_C_/F (L) | 8.32 (116%) |  | 9.09 | (4.92, 37.6) |
|  | Ka (h^-1^) | 0.773 (12.0%) |  | 0.790 | (0.592, 1.18) |
|  | V_T_/F (L) | 326 (19.9%) |  | 322 | (216, 700) |
|  | Q/F (L/h) | 44.1 (16.2%) |  | 46.4 | (30.7, 78.4) |
| **covariates** | *Salmonella* on CL/F | 0.124 (32.7%) |  | 0.128 | (0.0406, 0.367) |
| **IIV** | $\omega^{2}$ (CL/F) | 0.102 (44.3%) | 15.6% | 0.0894 | (0.00849, 0.176) |
| **RV** | $\sigma^{2}$ | 0.193 (17.4%) | 5.50% | 0.182 | (0.109, 0.295) |
| **2) DAB (P-gp)** | | | | Minimization successful: 98% | |
| **structural model** | CL/F (L/h) | 150 (6.10%) | - | 147 | (108, 162) |
|  | V_C_/F (L) | 865 (14.1%) |  | 851 | (208, 1061) |
|  | Ka (h^-1^) | 1.37 (20.6%) |  | 1.38 | (0.317, 1.94) |
|  | V_T_/F (L) | 535 (25.4%) |  | 605 | (303, 5000) |
|  | Q/F (L/h) | 66.2 (37.9%) |  | 70.2 | (41.3, 116) |
| **covariates** | Type 2 diabetes on F | 1.23 (7.2%) |  | 1.24 | (1.03, 1.49) |
|  | Urea on CL/F | -0.749 (21.5%) |  | -0.758 | (-1.23, -0.475) |
| **IIV** | $\omega^{2}$ (CL/F) | 0.0459 (35.1%) | 5.80% | 0.0448 | (0.01697, 0.0950) |
|  | $\omega^{2}$ (VC/F) | 0.0556 (40.8%) | 14.7% | 0.0575 | (0.0222, 0.110) |
| **RV** | $\sigma^{2}$ | 0.0391 (16.7%) | 15.7% | 0.0363 | (0.0246, 0.0465) |
| **3) PTV (OATP)** | | | | Minimization successful: 98% | |
| **structural model** | CL/F (L/h) | 11.7 (23.5%) | - | 11.6 | (7.62, 14.4) |
|  | V_C_/F (L) | 12.2 (16.1%) |  | 12.1 | (8.84, 17.1) |
|  | F1 | 0.410 (6.60%) |  | 0.410 | (0.363, 0.444) |
|  | Ka (h^-1^) | 0.230 (13.1%) |  | 0.231 | (0.199, 0.307) |
|  | ALAG1 (h) | 0 FIX |  | 0 FIX | - |
|  | D2 (h) | 0.654 (6.90%) |  | 0.666 | (0.571, 0.740) |
|  | V_T_/F (L) | 378 (60.8%) |  | 378 | (178, 821) |
|  | Q/F (L/h) | 10.9 (18.2%) |  | 11.0 | (8.96, 14.2) |
| **covariates** | Male on CL/F | 1.42 (14.5%) |  | 1.45 | (1.19, 1.84) |
| **IIV** | $\omega^{2}$ (CL/F) | 0.078 (54.9%) | 7.00% | 0.0726 | (0.0386, 0.150) |
|  | $\omega^{2}$ (VC/F) | 0.412 (33.7%) | 8.30% | 0.403 | (0.0818, 0.676) |
| **RV** | $\sigma^{2}$ | 0.0366 (16.6%) | 16.6% | 0.0337 | (0.0254, 0.0483) |
| **4) RSV (OATP, BCRP)** | | | | Minimization successful: 90% | |
| **structural model** | CL/F (L/h) | 66.9 (9.60%) | - | 66.2 | (46.2, 81.4) |
|  | V_C_/F (L) | 33.1 (25.4 %) |  | 34.5 | (13.0, 60.6) |
|  | F1 | 0.668 (9.00%) |  | 0.654 | (0.533, 0.772) |
|  | Ka (h^-1^) | 0.0651 (16.0%) |  | 0.0608 | (0.0317, 0.0791) |
|  | ALAG1 (h) | 0.88 (6.60%) |  | 0.866 | (0.694, 0.972) |
|  | D2 (h) | 7.3 (3.10%) |  | 7.28 | (6.62, 7.75) |
| **covariates** | BCRP_DecF_ on CL/F | 0.741 (12.3%) |  | 0.726 | (0.559, 0.977) |
|  | *Clostridium_XlVb* on CL/F | -0.0847 (32.8%) |  | -0.0828 | (-0.197, -0.0501) |
| **IIV** | $\omega^{2}$ (CL/F) | 0.134 (31.3%) | 3.30% | 0.112 | (0.0546, 0.185) |
|  | $\omega^{2}$ (VC/F) | 0.292 (53.4%) | 3.60% | 0.365 | (0.0828, 1.63) |
|  | $\omega^{2}$ (F1) | 0.615 (43.6%) | 10.0% | 0.531 | (0.0886, 1.31) |
| **RV** | $\sigma^{2}$ | 0.0695 (17.1%) | 16.8% | 0.0531 | (0.0264, 0.103) |
| **5) ATV (OATP, BCRP, P-gp, CYP3A)** | | | | Minimization successful: 85% | |
| **structural model** | CL/F (L/h) | 416 (11.0%) | - | 427 | (343, 525) |
|  | V_C_/F (L) | 4250 (17.6%) |  | 4104 | (2045, 5442) |
|  | D2 (h) | 0.500 (1.80%) |  | 0.500 | (0.437, 0.600) |
|  | V_T_/F (L) | 6050 (10.5%) |  | 6018 | (4878, 7178) |
|  | Q/F (L/h) | 3490 (12.0%) |  | 3643 | (3003, 4468) |
| **covariates** | Type 2 diabetes on F | 1.73 (9.00%) |  | 1.76 | (1.45, 2.08) |
| **IIV** | $\omega^{2}$ (CL/F) | 0.0502 (80.5%) | 37.8% | 0.0424 | (0.00428, 0.1351) |
|  | $\omega^{2}$ (VC/F) | 0.541 (36.6%) | 12.6% | 0.558 | (0.206, 0.771) |
| **RV** | $\sigma^{2}$ | 0.0878 (17.0%) | 9.50% | 0.0866 | (0.0686, 0.103) |

CL/F, apparent clearance; V_C_/F, apparent volume of distribution for the central compartment; V_T_/F, apparent volume of distribution for the peripheral compartment; Q/F, apparent clearance between V_C_/F and V_T_/F; Ka, first-order absorption rate constant; F1, fraction of dose absorbed by first-order absorption; ALAG1, lag time of first-order absorption; D2, duration of zero-order absorption; BCRP_DecF_, participants with decreased function (DecF) of BCRP; IIV, interindividual variabilities; RV, proportional residual variation.

**Table S5.** Gene polymorphisms frequencies (%) across study population.

| **DMET** | **SNP** | **Genotype** | **HVs**  **No. (%) of participants** | **Type 2 diabetes**  **No. (%) of participants** |
| --- | --- | --- | --- | --- |
| CYP3A4 | rs2242480 | CC | 6 (46.2 %) | 8 (61.5 %) |
|  |  | TT | 3 (23.1 %) | 1 (7.69 %) |
|  |  | TC | 4 (30.7 %) | 4 (30.8 %) |
|  | rs3735451 | CC | 3 (25 %) | 1 (7.69 %) |
|  |  | CT | 5 (41.7 %) | 5 (38.5 %) |
|  |  | TT | 4 (33.3 %) | 7 (53.8 %) |
| CYP3A5 | rs776746 | TT | 2 (15.4 %) | 1 (7.69 %) |
|  |  | TC | 7 (53.8 %) | 4 (30.8 %) |
|  |  | CC | 4 (30.8 %) | 8 (61.5 %) |
| P-gp | rs1045642 | AA | 0 (0%) | 2 (15.4 %) |
|  |  | AG | 6 (46.2 %) | 8 (61.5 %) |
|  |  | GG | 7 (53.8 %) | 3 (23.1 %) |
|  | rs1128503 | AA | 7 (50 %) | 5 (38.5 %) |
|  |  | GG | 3 (21.4 %) | 1 (7.69 %) |
|  |  | GA | 4 (28.6 %) | 7 (53.8 %) |
|  | rs2032582 | AA | 0 (0 %) | 1 (7.69 %) |
|  |  | AT | 1 (7.14 %) | 1 (7.69 %) |
|  |  | CC | 5 (35.7 %) | 2 (15.4 %) |
|  |  | CA | 6 (42.9 %) | 7 (53.8 %) |
|  |  | CT | 0 (0 %) | 2 (15.4 %) |
|  |  | TT | 2 (14.3 %) | 0 (0 %) |
| OATP1B1 | rs2306283 | AA | 1 (7.14 %) | 0 (0 %) |
|  |  | AG | 3 (21.4 %) | 7 (53.8 %) |
|  |  | GG | 10 (71.4 %) | 6 (46.2 %) |
|  | rs4149056 | CC | 1 (7.14 %) | 0 (0 %) |
|  |  | TT | 11 (78.6 %) | 10 (76.9 %) |
|  |  | TC | 2 (14.3 %) | 3 (23.1 %) |
| OATP1B3 | rs4149117 | GG | 7 (53.8 %) | 8 (61.5 %) |
|  |  | GT | 5 (38.5 %) | 5 (38.5 %) |
|  |  | TT | 1 (7.69 %) | 0 (0 %) |
| BCRP | rs2231142 | GG | 6 (46.2 %) | 8 (61.5 %) |
|  |  | GT | 7 (53.8 %) | 5 (38.5 %) |

**Table S6.** Genotype-predicted phenotype status across study groups.

| **DMET** | **Phenotype** | **HVs**  **No. (%) of participants** | **Type 2 diabetes**  **No. (%) of participants** | ***P* value** |
| --- | --- | --- | --- | --- |
| CYP3A5 | NM / IM | 9 (69.2 %) | 5 (38.5 %) | 0.116 |
|  | PM | 4 (30.8 %) | 8 (61.5 %) |  |
| OATP1B1 | NF | 11 (78.6 %) | 10 (76.9 %) | 0.918 |
|  | PF / DecF | 3 (21.4 %) | 3 (23.1 %) |  |
| BCRP | NF | 6 (46.2 %) | 8 (61.5 %) | 0.431 |
|  | DecF | 7 (53.8 %) | 5 (38.5 %) |  |

**Table S7.** Demographic and clinical characteristics of the two groups after balancing age and body weight between them.

| **Parameters** | **HV (N=10)** | **Type 2 diabetes (N=13)** | ***P* value** |
| --- | --- | --- | --- |
| Age (years) | 40.90 ± 11.91 | 49.23 ± 11.58 | 0.106 |
| Female: Male, No. (%) | 7: 3, (70.0% :30.0%) | 8: 5, (61.5%: 38.5 %) | 1.00 |
| Weight (kg) | 58.90 ± 7.23 | 67.08 ± 9.96 | 0.059 |
| BMI (kg/m^2^) | 22.91 ± 2.32 | 24.90 ± 2.22 | 0.064 |
| HbA1c (%) | ND | 7.62 ± 0.77 | NA |
| ALT (U/L) | 14.40 ± 5.25 | 25.23 ± 13.92 | 0.020* |
| AST (U/L) | 19.40 ± 5.40 | 23.77 ± 11.21 | 0.234 |
| ALB (g/L) | 43.80 ± 3.07 | 45.82 ± 2.61 | 0.103 |
| Urea (mmol/L) | 4.82 ± 1.07 | 4.72 ± 1.60 | 0.860 |
| Time since diagnostic  (years) | 0 | 2.62 ± 1.99 | NA |

Continuous variables are presented as mean ± SD. *Demographic parameters are significantly different between study groups (*P* < 0.05). HV, Healthy volunteer group; BMI, body mass index; HbA1c, glycated hemoglobin; ALT, alanine aminotransferase; AST, aspartate aminotransferase; ALB, serum albumin; ND, not detected; NA, not applicable.

**Table S8.** PK parameters were calculated at the intersecting time points between the HV and type 2 diabetes groups after balancing age and body weight between them.

| **Probe drugs**  **(DMET)** | **Parameters (Unit)** | **HV (N=10)** | **Type 2 diabetes (N=13)** | **Type 2 diabetes/HV** | ***P* value** |
| --- | --- | --- | --- | --- | --- |
| MDZ  (CYP3A) | C_max_ (pg/mL) | 40.5 (52.2%) | 55.7 (26.2%) | 1.38 | 0.054^#^ |
|  | AUC_0-24 h_ (h·pg/mL) | 123 (61.9%) | 138 (60.7%) | 1.12 | 0.649 |
| DAB  (P-gp) | C_max_ (pg/mL) | 304 (33.4%) | 361 (29.5%) | 1.18 | 0.212 |
|  | AUC_0-24 h_ (h·pg/mL) | 2224 (62.7%) | 3242 (38.8%) | 1.45 | 0.081^#^ |
| PTV  (OATP) | C_max_ (pg/mL) | 155 (37.8%) | 147 (50.5%) | 0.948 | 0.779 |
|  | AUC_0-24 h_ (h·pg/mL) | 496 (30.8 %) | 513 (38.9%) | 1.03 | 0.826 |
| RSV  (OATP, BCRP) | C_max_ (pg/mL) | 72.4 (55.4%) | 81.2 (75.1%) | 1.12 | 0.695 |
|  | AUC_0-24 h_ (h·pg/mL) | 609 (73.0 %) | 886 (64.4%) | 1.45 | 0.220 |
| ATV  (OATP, BCRP, P-gp, CYP3A) | C_max_ (pg/mL) | 14.9 (37.3%) | 22.7 (51.4%) | 1.52 | 0.048* |
|  | AUC_0-24 h_ (h·pg/mL) | 130 (37.8%) | 214 (48.6%) | 1.65 | 0.020* |

DMET, drug-metabolic enzymes and transporters; Type 2 diabetes/HV: ratio of PK parameters for type 2 diabetes patients versus HVs. PK parameters are presented as mean (CV%). *PK parameters are significantly different between study groups (*P* < 0.05). ^#^*P* < 0.1.
